# Assessment of associations between inhaled formaldehyde and lymphohematopoietic cancer through the integration of epidemiological and toxicological evidence with biological plausibility

**DOI:** 10.1093/toxsci/kfae039

**Published:** 2024-03-28

**Authors:** Melissa J Vincent, Seneca Fitch, Lauren Bylsma, Chad Thompson, Sarah Rogers, Janice Britt, Daniele Wikoff

**Affiliations:** ToxStrategies, LLC, Asheville, North Carolina 28801, United States; ToxStrategies, LLC, Asheville, North Carolina 28801, United States; EpidStrategies, a Division of ToxStrategies, LLC, Katy, Texas 77494, United States; ToxStrategies, LLC, Katy, Texas 77494, United States; ToxStrategies, LLC, Asheville, North Carolina 28801, United States; ToxStrategies, LLC, Asheville, North Carolina 28801, United States; ToxStrategies, LLC, Asheville, North Carolina 28801, United States

**Keywords:** formaldehyde, leukemia, lymphoma, risk of bias, systematic review, evidence integration

## Abstract

Formaldehyde is recognized as carcinogenic for the portal of entry sites, though conclusions are mixed regarding lymphohematopoietic (LHP) cancers. This systematic review assesses the likelihood of a causal relationship between formaldehyde and LHP cancers by integrating components recommended by NASEM. Four experimental rodent bioassays and 16 observational studies in humans were included following the implementation of the *a priori* protocol. All studies were assessed for risk of bias (RoB), and meta-analyses were conducted on epidemiological studies, followed by a structured assessment of causation based on GRADE and Bradford Hill. RoB analysis identified systemic limitations precluding confidence in the epidemiological evidence due to inadequate characterization of formaldehyde exposure and a failure to adequately adjust for confounders or effect modifiers, thus suggesting that effect estimates are likely to be impacted by systemic bias. Mixed findings were reported in individual studies; meta-analyses did not identify significant associations between formaldehyde inhalation (when measured as ever/never exposure) and LHP outcomes, with meta-SMRs ranging from 0.50 to 1.51, depending on LHP subtype. No associations with LHP-related lesions were reported in reliable animal bioassays. No biologically plausible explanation linking the inhalation of FA and LHP was identified, supported primarily by the lack of systemic distribution and *in vivo* genotoxicity. In conclusion, the inconsistent associations reported in a subset of the evidence were not considered causal when integrated with the totality of the epidemiological evidence, toxicological data, and considerations of biological plausibility. The impact of systemic biases identified herein could be quantitatively assessed to better inform causality and use in risk assessment.

Formaldehyde is a volatile organic compound that is ubiquitous in the general environment and is produced by both natural (eg, bacteria, plants, and animals) and anthropogenic sources (eg, building materials, tobacco smoke, consumer products, medications, automobile exhaust, and manufacturing facilities) (ATSDR, 2008). Formaldehyde is also an essential, naturally occurring metabolic intermediate in animals and humans; it is endogenously produced via serine, glycine, methionine, and choline metabolism and via the demethylation of N-, S-, and O-methyl compounds (USEPA, 2008) and then reutilized to make other hydrocarbon-containing molecules (such as purines, thymidine, and certain amino acids) in the body (Owen *et al.*, 1990).

Although it has a natural role as a metabolic intermediate, formaldehyde is considered a carcinogen by multiple authoritative agencies, including the USEPA (2022), primarily based on evidence of nasal tumors in rats and nasopharyngeal and sinonasal cancers in humans. However, the role of formaldehyde in the formation of other cancers, including lymphohematopoietic (LHP) cancers, is less well-established. Mixed conclusions have been offered among individual studies as well as reviews and meta-analyses of epidemiological data evaluating the associations between inhaled formaldehyde and LHPs (Bachand *et al.*, 2010; Catalani *et al.*, 2019; Checkoway *et al.*, 2012; Collins and Lineker, 2004; La Torre *et al.*, 2023; Mundt *et al.*, 2018, 2021; Protano *et al.*, 2022; Schwilk *et al.*, 2010; Zhang *et al.*, 2009). Many of these reviews evaluated associations between formaldehyde and leukemias, in general, taking varied approaches to combining LHPs (despite their etiological diversity). Moreover, these reviews largely lack a formal evaluation of causation; none of the available reviews fully employ systematic review methodology, including the use of *a priori* protocol development, clearly defined inclusion and exclusion criteria, critical appraisal of study quality through risk of bias (RoB) assessment, and use formal integration methods to assess causal relationships.

Recent authoritative assessments have also investigated formaldehyde exposure in relation to LHPs; however, these assessments have also not fully employed systematic review methodology and causation assessment. For example, the most recent draft IRIS assessment by USEPA (2022) states that “the available epidemiological studies provide *robust* evidence of an association consistent with causation between formaldehyde exposure and increased risk of myeloid leukemia,” “slight evidence” for a causal association with multiple myeloma and Hodgkin lymphoma, and “indeterminate evidence” to support an association between lymphatic leukemia and formaldehyde inhalation. However, these categorizations were not determined using formal consideration of causality, including consideration of biological plausibility or experiment; specifically, the USEPA was not able to identify a plausible mode of action (MOA) for leukemogenesis, lymphomagenesis, or myelomagenesis. In the subsequent review by the National Academy of Sciences, experts critiqued the USEPA’s assessment is based solely on evidence in humans and does not adequately incorporate tools for systematic review, RoB, or study quality evaluation (NASEM, 2023). The USEPA does not adequately address the lack of concordance between human and animal evidence and has not established a mode of action to support a causal association between formaldehyde inhalation and LHPs, including myeloid leukemia (NTP, 2021; USEPA, 2022). Specifically, NASEM (2023) concluded that the USEPA’s Population, Exposure, Comparator, and Outcome (ie, PECO) statements were not sufficiently clear or rationalized, and evidence synthesis and integration methods, as well as terminology, were not standardized or consistent with current USEPA guidance or international practice. Although NASEM’s comments were specific to USEPA’s assessment, they apply to other reviews and integrations of evidence that have been published, to date. Despite the number of reviews available, none—including that from the USEPA, have both (1) fully applied systematic review methodology (including critical appraisal via RoB) and (2) fully considered the integration of epidemiological information with toxicological and mode of action information and biological plausibility.

Further complicating the regulatory landscape, there is no scientific consensus regarding the causal basis of the mixed associations between exposure and LHP cancers observed across studies. NTP (2021) states that epidemiological evidence supports a causal relationship between formaldehyde inhalation and cancer in humans, however, other reviews have noted that observed associations between formaldehyde and LHPs in the epidemiological literature may be attributable to chance, bias, or confounding and that the cumulative weight of evidence is not supportive of causality (Allegra *et al.*, 2019; Andersen *et al.*, 2019; Checkoway *et al.*, 2012; Gentry *et al.*, 2020; Mundt *et al.*, 2018; Protano *et al.*, 2022; Rhomberg *et al.*, 2011). Most recently, the USEPA IRIS program asserted that there is a causal relationship between formaldehyde and nasopharyngeal, sinonasal, and some LHP cancers, including myeloid leukemia, multiple myeloma, and Hodgkin lymphoma.

Therefore, despite published meta-analyses that identified a potential hazard based on significant increases in LHPs associated with formaldehyde inhalation, uncertainty remains regarding the observed associations and their utility in evaluating whether the observed associations are casual in nature. These reviews and meta-analyses rely on the same underlying epidemiological and toxicological evidence, yet they have come to differing conclusions regarding the weight of evidence regarding causal associations between LHPs and formaldehyde inhalation. As such, the objective herein is the conduct of a systematic review using methods consistent with international practice to assess the potential causal relationship between formaldehyde exposure and LHPs. Emphasis is placed on the critical appraisal of epidemiological data and integrated consideration of experimental animal and mechanistic evidence streams to inform the assessment of causality. This review specifically addresses NASEM (2023) comments regarding the limitations in draft USEPA (2022) systematic review and evidence integration. It is anticipated that this review will provide transparency in the development of conclusions, as well as to demonstrate the importance of appropriately applying critical appraisal tools and considering biological plausibility in systematic reviews facilitating risk assessment.

## Materials and methods

The analysis of a causal relationship between inhaled formaldehyde and LHP cancers was facilitated by systematic review methodology consistent with international recommendations (NTP, 2015; WHO, 2021). Conduct and reporting is aligned with AMSTAR2 (a critical appraisal tool for systematic reviews) as well as both PRISMA-P (protocol reporting checklist) and PRISMA (systematic review and meta-analyses reporting checklist), and Meta-analysis of Observational Studies in Epidemiology guidelines (Brooke *et al.*, 2021; Page *et al.*, 2021; Shea *et al.*, 2017). A PRISMA-P compliant protocol was developed *a priori* based on scoping and problem formulation exercises and posted publicly to the Center for Open Science’s Open Science Framework, an online data repository (Fitch *et al.*, 2023), which can be accessed at: https://osf.io/dqasf. Detailed methodology related to the assessment methods is reported therein, and briefly described here.

The PECO, or research question, “Is there a causal relationship between inhaled formaldehyde and lymphohematopoietic cancer in humans?” was established; search terms were developed and utilized per the protocol (Supplementary Material 1; Fitch *et al.*, 2023). Eligibility criteria are described in the protocol (Fitch *et al.,* 2023); briefly, these criteria include primary epidemiological and mammalian toxicological literature in which inhaled formaldehyde is the primary exposure (including formalin) and the incidence or mortality of specific LHP malignancies (ie, Hodgkin lymphoma, multiple myeloma, lymphoid leukemia, myeloid leukemia, or monocytic leukemia) are measured as the primary outcome; syntheses of mode of action information were retained and used for assessment of biological plausibility.

### Evidence identification and data extraction

The search strategy was developed by an information specialist and validated by the assessment team. The strategy relied on 3 sources: handsearching and reference chasing from existing reviews and meta-analysis, reference chasing from authoritative assessments, and independent search of the peer-reviewed literature using 2 databases (PubMed and Embase) based on citation-specific search syntax (see Supplementary Material 1 and protocol; Fitch *et al.*, 2023). For development of the citation database search syntax, the assessment team used key words related to “formaldehyde” and LHP outcomes. Additionally, USEPA’s recent Toxicological Report on Formaldehyde (2022) was consulted for additional relevant terms. The literature searches by the USEPA (2022) were deemed “adequate” and consistent with NASEM guidance (2023). Any related terms identified to be missing were incorporated in the syntax for this assessment. To validate the performance of this search, reviewers compared studies identified as relevant during scoping and problem formulation to the results of the search. Citations were deduplicated using EndNote and DistillerSR. Subsequent title/abstract screening, full-text screening, data extraction, and critical appraisal were conducted in DistillerSR. Forms specific to each stage of evidence identification were developed by an information specialist and piloted by the assessment team. For title/abstract screening and full-text review, 2 reviewers screened each citation. Disagreements between reviewers were resolved by a third, senior reviewer (D.W.). If the citation met inclusion criteria, it was advanced to data extraction and the RoB assessment. This step was performed by a single reviewer using project-specific forms in DistillerSR, and confirmed with a 100% QC by a second reviewer. To account for updated cohort analyses, DistillerSR’s “Linked Paper” function was utilized during the full-text review. Each citation reporting epidemiological data was tagged with the assessed cohort name. Only the most recent analysis of the cohort was retained for use in meta-analyses.

### Critical appraisal via risk of bias assessment

Following data extraction, NTP OHAT’s Risk of Bias Tool for Human and Animal Studies (2015) was used to assess internal validity via RoB for human and experimental animal evidence streams. This approach includes evaluating individual studies based on 10 questions that interrogate internal validity, though not all questions are relevant to both human and experimental animal study designs. For each question, the reviewer rated the study with one of 4 levels of potential risk: “++” (definitely low RoB), “+” (probably low RoB), “−” (probably high RoB or not reported), “−−” (definitely high RoB). When possible, reviewers referred to previous citations to inform each question for publications reporting cohort analysis updates.

During problem formulation and protocol development, NTP OHAT (2015) RoB guidance was reviewed for epidemiological and toxicological evidence assessment. For epidemiological studies, key questions were designated and specific guidance for each question was developed to address known formaldehyde-specific considerations as well as outcome-specific considerations (eg, risk factors evaluated as confounders). Although, as noted by Mundt *et al.* (2021), there are no common risk factors associated with all types of LHPs, critical confounders or covariates of interest include: age, sex, smoking status, and coexposures related to LHP risk (such as benzene, ionization radiation, and formalin or methanol use) (American Cancer Society, 2018a; Mundt *et al.*, 2018; Rhomberg *et al.*, 2011; USEPA, 2022). Ionizing radiation and formalin (methanol) exposures occur in populations of embalmers (Hauptmann *et al.*, 2009) and are known risk factors for myeloid leukemia. Methanol is metabolized to formaldehyde *in vivo* and may impact health outcomes or introduce uncertainty (USEPA, 2022). Race and obesity may also modify risks for some LHPs, such as multiple myeloma (American Cancer Society, 2018b). An overview of these refinements is provided in the Supplementary Table 1 and Fitch *et al.* (2023) and the detailed RoB assessment guidance (Supplementary Material 6). The assessment team determined the RoB guidance to be sufficient for toxicological evidence evaluation, and as such specific refinements were not made; however, general concepts of guideline-study design and conduct were utilized to guide the assessment of internal validity. The full guidance referenced by reviewers during the completion of the RoB tool is available in Supplementary Material. Relevant methodological information provided in prior publications or analyses for each cohort or population were considered when evaluating RoB.

Following completion of the RoB analysis, reviewer ratings for each study were arranged into a heat map and an overall rating of Tier 1, 2, or 3 was designated for each study based on guidance provided in NTP OHAT’s *Handbook for Conducting a Literature-Based Health Assessment* (NTP, 2019). Tiers are based on ratings across both key and supporting elements and required the following:

- **Tier 1:** A study is rated as “definitely low” or “probably low” RoB for key elements AND have a majority of other applicable items answered “definitely low” or “probably low” RoB.
- **Tier 2:** Study meets neither the criteria for first or third tiers.
- **Tier 3:** A study is rated as “definitely high” or “probably high” RoB for key elements AND have a majority of other applicable items answered “definitely high” or “probably high” RoB.

### Evidence synthesis and meta-analysis

Evidence from human and animal evidence streams were qualitatively synthesized by stream and subsequently integrated via structured assessment of causality and confidence in the body of evidence to assess such epidemiological data were also quantitatively synthesized via meta-analyses. For LHP subtypes with 3 or more unique and comparable effect estimates (eg, odds ratios and relative risks could be combined), fixed and random-effects meta-analyses were performed using the meta package in R (version 4.2.2). Random effects were estimated using the restricted maximum-likelihood estimator approach. Effect estimates were weighted by the inverse of their variance. Each LHP subtype was evaluated separately, consistent with the National Research Council’s (2011) recommendations to the USEPA, based on the understanding that LHPs are etiologically diverse (Mundt *et al.*, 2021).

Effect estimates used in the analysis included the natural log of the reported SMR, OR, or other relative risk to reduce bias through assumptions of normality in the underlying distributions. The standard error for log-transformed SMR estimates was calculated using the reported upper and lower confidence limits (equation 1).

(1) $${\text{SE}}_{\text{lnSMR}}=\text{ln}\left({\text{SMR}}_{\text{ucl}}/{\text{SMR}}_{\text{lcl}}\right)/3.92$$

In order to prevent repeated inclusion of the same groups of persons, only the most updated study for each cohort was included in the meta-analysis. For example, there are multiple studies evaluating the NCI cohort, and it is known that there is some overlap among studies of funeral directors and embalmers (Hayes *et al.*, 1990; Mundt *et al.*, 2018; Walrath and Fraumeni, 1983, 1984).

Some studies report an SMR of 0, based on 0 observed mortalities in the studied cohort (Stroup *et al.*, 1986). In order to estimate the lnSMR and SE_lnSMR_, an offset value of 0.01 was added to the SMR and the SMR_lcl_ of 0. Sensitivity analyses were conducted to evaluate the impact of offset value selection. If 95% CIs were not reported, then the SE was estimated as the inverse of the square root of the number of observed deaths.

Sensitivity analyses were conducted to evaluate the impact of inclusion or exclusion of studies with a comparatively higher RoB (eg, Tier III). Publication bias was evaluated using visual inspection of funnel plots and the Egger test for funnel plot asymmetry (see Supplementary Material 10).

### Evidence integration—assessment of causality

Epidemiological and experimental animal data were integrated and assessed both for the confidence in the body of evidence based on OHAT (and GRADE) and, further, to determine the potential for a causal relationship based on Bradford Hill (1965). It is of note that Bradford Hill considerations were designed to assess causation for observational evidence, whereas OHAT and GRADE are not. As such, the blended approach is whereby aspects specific to assessing causality are considered along with the structured confidence evaluation. Of particular importance, the structured integration considered biological plausibility based on toxicokinetics, mode of action, endogenous capacities, and genotoxicity. Hill considerations were used, recognizing their long-standing use and acceptance, including modifications which reflect the changing methods and standards in modern epidemiological and toxicological studies (Fedak *et al.*, 2015; Meek *et al.*, 2014; USEPA, 2005).

Collectively, the evaluation of causality involved structured assessment of: (1) RoB, including residual and confounding bias; (2) consistency within evidence streams; (3) publication bias; (4) strength of association; (5) specificity of the exposure-response association or essentiality of MOA key events; (6) temporality of the exposure-response relationship and observed key events; (7) exposure-response gradient; (8) biological plausibility; (9) coherence among evidence streams; (10) analogy, or evidence of causation from a similar causal agent (eg, other aldehydes); (11) imprecision; and (12) indirectness, or lack of applicability within both the epidemiological and toxicological evidence. The criterion of “experiment” is considered for toxicological evidence, but not for epidemiological information in this analysis, as LHPs have a variable and potentially long latency period of approximately 2–35 years, depending on the LHP type (World Trade Center Health Program, 2014); cessation of exposure may not directly result in a cessation of effect.

Confidence in the body of evidence was assessed using NTP OHATs confidence ratings (a modification of the GRADE criteria) (Table 1); this exercise was specific to assessing confidence in available data, recognizing that the GRADE criteria overlap with Bradford Hill, but are not alone sufficient for assessment of causality due to the lack of consideration for biological plausibility or evidence integration. GRADE was also not considered sufficient due to limitations in applicability to risk assessment and evaluation of adverse effects using studies other than controlled trials in humans.

**Table 1.** Methods for assessing confidence in the body of evidence and causality based both on the Bradford Hill criteria (bold, underlined) and NTP OHAT (2015) (based on GRADE)

Initial confidence of key features of study design*^a^*	Factors influencing confidence in the body of evidence (based on OHAT and GRADE)—increase or decrease	Additional factors influencing assessment of causal relationship Increase or decrease
High (++++)	Risk of bias within and across studies and evidence streams; impact (or estimated magnitude) and direction of biases or residual confounding Imprecision in effect estimates within and across studies and evidence streams Consistency within and across evidence streams; ability to explain inconsistencies Magnitude of effect (**strength**) Presence and consistency of exposure/dose-response **(biological gradient)** Directness of the available research for addressing the research question Publication bias	*[All factors associated with confidence in body of evidence]* **Biological plausibility** (mode of action, toxicokinetics) **Experiment** **Coherence** within and across evidence streams based on an integrated consideration of consistency in direction and magnitude of effect (or lack of effect) within and across evidence streams, including exposure-response gradient **Temporality** **Analogy** **Specificity**
Moderate (+++)		
Low (++)		
Very low (+)		

a Initial confidence is generally defined by the number of study features: including controlled exposure, exposure occurring prior to the outcome, use of individual outcome data, and use of a comparison group. “High” has 4 features, “Moderate” has 3, “Low” has 2, and “Very Low” has ≤1.

## Results

###  

#### Summary of literature identification, selection, and extraction

**Figure 1.**
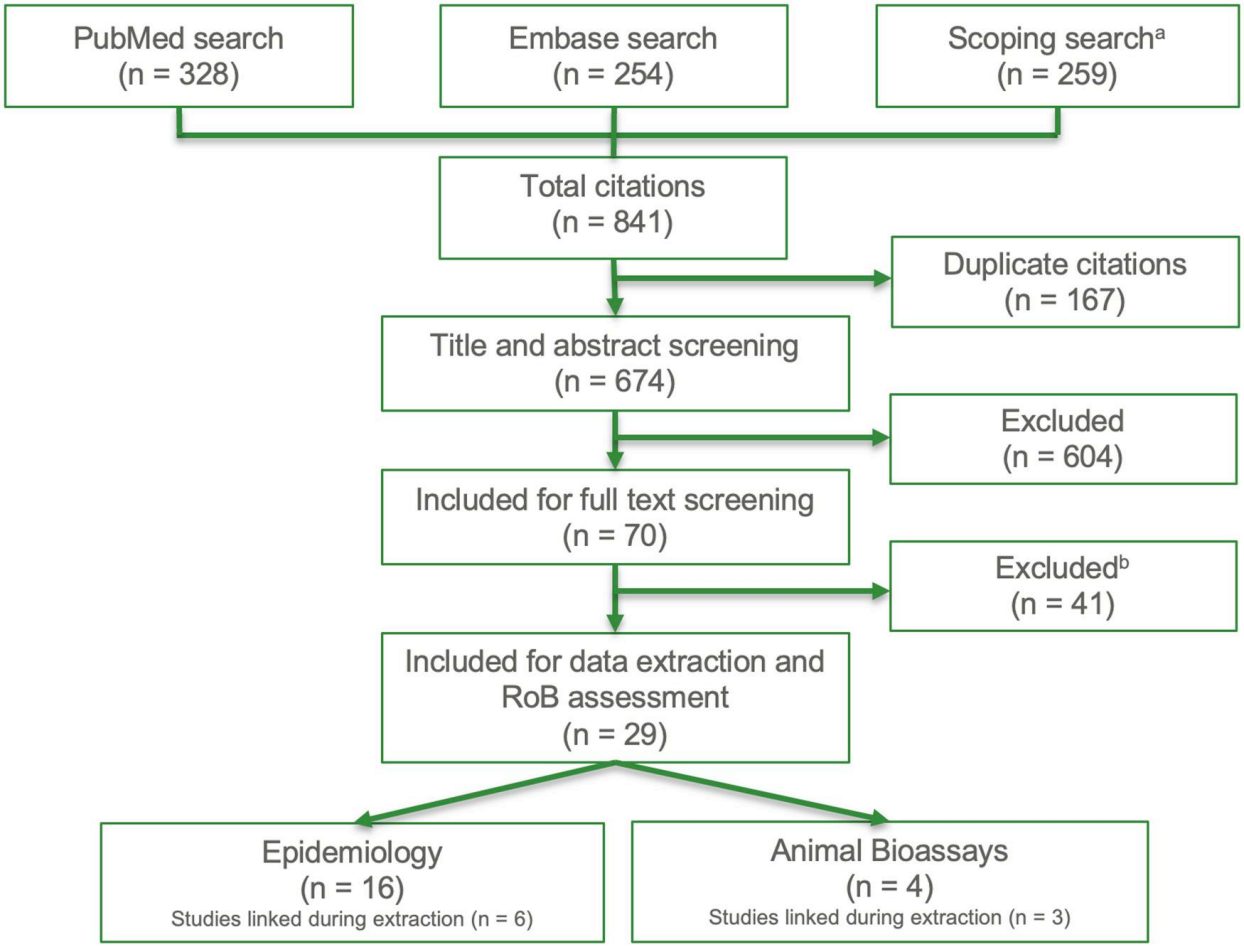
Literature search and review results. ^*a*^The scoping search was a hand search of multiple reviews, meta-analyses, and government agency reports and their bibliographies to ensure the completeness of the database. ^*b*^Following full-text review, a total of reasons for exclusion of studies at the full-text level (*n* = 41) include: no LHP outcome, review/meta-analysis, *in vitro* or nonmammalian assay, formaldehyde not primary exposure, no unexposed control, case study/series, nonspecific LHP outcome or non-Hodgkin’s lymphoma, noninhalational exposure route (see Supplemental Material for the complete list of excluded studies).

Following the systematic search of the literature, 29 studies were included in this assessment, including 16 epidemiological studies and 4 experimental animal studies (Figure 1; see Supplementary Material 2 for a complete list of excluded studies). Six of the identified cohort studies were updated in more recent analyses, and 2 publications were identified to report portions of a complete study report. These studies were linked to the complete study reports or updated cohort analyses (see Supplementary Material 3). Two publications reported similar analyses of the NCI formaldehyde cohort but were ultimately not linked as they were identified as independent analyses (Beane Freeman *et al.*, 2009; Checkoway *et al.*, 2015). Study details were extracted for all included studies and provided in Supplementary Materials 4 and 5.

#### Assessment and synthesis of epidemiological evidence

Across the 16 epidemiological studies included, LHPs were investigated in numerous cohorts: garment workers (Meyers *et al.*, 2013), UK formaldehyde users and producers (Coggon *et al.*, 2014), the National Cancer Institute (NCI) cohort (Beane Freeman *et al.*, 2009; Checkoway *et al.*, 2015), embalmers, undertakers, and funeral directors (Hauptmann *et al.*, 2009; Hayes *et al.*, 1990; Linos *et al.*, 1990; Walrath and Fraumeni, 1983, 1984); U.K. and U.S. Pathologists (Hall *et al.*, 1991; Matanoski, 1989), Members of the American Association of Anatomists (Stroup *et al.*, 1986), Iron Foundry workers (Andjelkovich *et al.*, 1995), U.S. Chemical Manufacturing Workers (Ott *et al.*, 1989); Laminated Plastic Workers (Pira *et al.*, 2014), and LHP cancer patients matched with population controls (Gérin *et al.*, 1989). Associations between inhaled formaldehyde exposure and LHP subtypes of Hodgkin lymphoma, multiple myeloma, myeloid leukemia, lymphoid leukemia, and monocytic leukemia were investigated (Table 2; see Supplementary Material 4 for tabular summaries of individual studies).

**Table 2.** Summary of identified epidemiological evidence for each LHP cancer subtype

LHP cancer subtype	**Cohorts** *^a^*	**Studies (RefID)** *^b^*	Study designs	Reported effect estimates	Outcome conclusion based on qualitative and quantitative synthesis (and including risk of bias)
Hodgkin lymphoma	NCI; embalmers licensed in New York and California; members of the American Association of Anatomists; U.S. embalmers and funeral directors; RCPath members; U.S. garment workers; iron foundry workers; LHP cancer patient cases matched with population controls; U.S. pathologists	Andjelkovich *et al.*, 1995; Beane Freeman *et al.*, 2009; Checkoway *et al.*, 2015; Gérin *et al.*, 1989; Hall *et al.*, 1991; Hayes *et al.*, 1990; Matanoski, 1989; Meyers *et al.*, 2013; Stroup *et al.*, 1986; Walrath and Fraumeni, 1983, 1984	Cohort and case-control	SMR (ever/never exposure; Andjelkovich *et al.*, 1995; Beane Freeman *et al.*, 2009; Checkoway *et al.*, 2015; Hall *et al.*, 1991; Matanoski, 1989; Meyers *et al.*, 2013; Stroup *et al.*, 1986) RR (striation by average, peak, and cumulative exposure estimations; Beane Freeman *et al.*, 2009) PMR (Hayes *et al.*, 1990; Walrath and Fraumeni, 1983, 1984) HR (striation by cumulative and peak exposures; Checkoway *et al.*, 2015) OR (ever/never exposure; Gérin *et al.*, 1989)	Sufficient information for meta-analysis of SMRs based on ever/never exposures, but insufficient information for pooled OR/RR/HR estimates based on groupings of exposure No statistically significant evidence of association of Hodgkin lymphoma with ever/never exposure; exposure-response trends reported by Beane Freeman *et al.* (2009) and Checkoway *et al.* (2015) in the NCI cohort based on increasing peak and cumulative exposures
Monocytic leukemia	Embalmers licensed in New York and California	Walrath and Fraumeni, 1983, 1984	Retrospective cohort	Ever/never: 2 PMR (Walrath and Fraumeni, 1983, 1984)	Limited evidence—mixed evidence; insufficient information for meta-analysis
Lymphoid leukemia	NCI; embalmers licensed in NY and California; U.S. embalmers and funeral directors; U.S. garment workers; U.S. Chemical manufacturing workers	Beane Freeman *et al.*, 2009; Checkoway *et al.*, 2015; Hayes *et al.*, 1990; Meyers *et al.*, 2013; Ott *et al.*, 1989; Walrath and Fraumeni, 1984	Cohort and nested case-control	SMR (ever/never exposure; Beane Freeman *et al.*, 2009; Checkoway *et al.*, 2015; Meyers *et al.*, 2013) PMR (Hayes *et al.*, 1990; Walrath and Fraumeni, 1984) RR (striation by average, peak, and cumulative exposure estimations; Beane Freeman *et al.*, 2009) HR (striation by cumulative and peak exposures; Checkoway *et al.*, 2015) OR (ever/never exposure; Ott *et al.*, 1989)	Insufficient information for meta-analysis No statistically significant evidence of association of lymphoid leukemia with ever/never exposure; exposure-response trends reported by Beane Freeman *et al.* (2009) and Checkoway *et al.* (2015) in the NCI cohort were not statistically significant
Multiple myeloma	NCI; U.S. embalmers and funeral directors; British chemical workers; Laminated plastic factory workers; U.S. garment workers; U.S. chemical manufacturing workers	Beane Freeman *et al.*, 2009; Checkoway *et al.*, 2015; Coggon *et al.*, 2014; Hayes *et al.*, 1990; Meyers *et al.*, 2013; Ott *et al.*, 1989; Pira *et al.*, 2014	Cohort and nested case-control	SMR (ever/never exposure: Beane Freeman *et al.*, 2009; Checkoway *et al.*, 2015; Coggon *et al.*, 2014; Meyers *et al.*, 2013; Pira *et al.*, 2014; striation by peak exposure: Coggon *et al.*, 2014; striation by duration or year of exposure: Meyers *et al.*, 2013) PMR (Hayes *et al.*, 1990) RR (striation by average, peak, and cumulative exposure estimations; Beane Freeman *et al.*, 2009) HR (striation by cumulative and peak exposures; Checkoway *et al.*, 2015) OR (ever/never exposure; Ott *et al.*, 1989)	Sufficient information for meta-analysis of SMRs based on ever/never exposures Available information does not support association of multiple myeloma with ever/never exposure*^c^*; exposure-response trends reported by Beane Freeman *et al.* (2009) and Checkoway *et al.* (2015) in the NCI cohort were not statistically significant; no statistically significant impact of duration or year of exposure (Meyers *et al.*, 2013) Insufficient information for pooled OR/RR/HR estimates based on groupings of exposure
Myeloid leukemia	NCI; U.S. funeral industry; embalmers licensed in New York and California; members of the American Association of Anatomists; U.S. embalmers and funeral directors; embalmers and funeral directors of Minnesota and Iowa; British chemical workers; U.S. garment workers; U.S. chemical manufacturing workers	Beane Freeman *et al.*, 2009; Checkoway *et al.*, 2015; Coggon *et al.*, 2014; Hauptmann *et al.*, 2009; Hayes *et al.*, 1990; Linos *et al.*, 1990; Meyers *et al.*, 2013; Ott *et al.*, 1989; Stroup *et al.*, 1986; Walrath and Fraumeni, 1983, 1984	Cohort, case-control, and nested case-control	SMR (ever/never exposure: Beane Freeman *et al.*, 2009; Checkoway *et al.*, 2015; Coggon *et al.*, 2014; Meyers *et al.*, 2013; Stroup *et al.*, 1986; striation by peak exposure: Coggon *et al.*, 2014; striation by duration or year of exposure Meyers *et al.*, 2013) PMR (Hayes *et al.*, 1990; Walrath and Fraumeni, 1983, 1984)*^d^* RR by age of exposure (Meyers *et al.*, 2013) and striation by average, peak, and cumulative exposure estimations; Beane Freeman *et al.*, 2009) HR (striation by cumulative and peak exposures; Checkoway *et al.*, 2015) OR (ever/never exposure: Linos *et al.*, 1990; Ott *et al.*, 1989; striated by duration of exposure: Meyers *et al.*, 2013; and average exposure: Coggon *et al.*, 2014; Hauptmann *et al.*, 2009; and peak, and cumulative exposure: Hauptmann *et al.*, 2009) SRR by duration of exposure (Meyers *et al.*, 2013)	Sufficient information for meta-analysis of SMRs based on ever/never exposures Available information does not support association with myeloid leukemia and ever/never exposure, as measured using SMR. Insufficient information for pooled OR/RR/HR estimates based on groupings of exposure Mixed evidence regarding duration of exposure or cumulative exposure (Beane Freeman *et al.*, 2009; Hauptmann *et al.*, 2009; Coggon *et al.*, 2014; Checkoway *et al.*, 2015; Meyers *et al.*, 2013) or intensity of exposure (measured as peak exposure) (Beane Freeman *et al.*, 2009; Hauptmann *et al.*, 2009; Stroup *et al.*, 1986; Checkoway *et al.*, 2015); most trends are not statistically significant and do not show significantly increased risk (Checkoway *et al.*, 2015, as an example). However, some trends are statistically significant (eg, Hauptmann *et al.*, 2009)

a Some cohorts are known to overlap, including the embalmers and funeral directors (Hauptmann *et al.* 2009; Walrath and Fraumeni, 1983, 1984).

b Blair *et al.* (1986) is an earlier study of the NCI Cohort; Beane Freeman *et al.* (2009) and Checkoway *et al.* (2015) are the most recent updates, with Beane Freeman *et al.* (2009) being the update and Checkoway *et al.* (2015), a re-analysis of the updated cohort.

c Hayes *et al.* (1990) reports a PMR of 369 (95% CI 159–726) among nonwhites, however, the sample size is comparatively small (*N* = 397), is inconsistent with other reported information, and may be subject to bias.

d Only Hayes *et al.* (1990) report statistical significance; PMR 157 (95% CI 101–234).

#####  

RoB assessment determined that each of the available epidemiological studies has at least one critical deficiency (Table 3). Each of the studies was rated as “probably high” or “definitely high” risk of detection bias due to limitations in exposure characterization. None of the identified studies measured exposure to formaldehyde; no studies provided robust, quantitative measurements of formaldehyde exposure. Studies that attempted to quantify exposure relied on historical recreations and an understanding of processes and job types to make determinations regarding peak or average exposures. This is critically important for occupational scenarios where formalin (a solution of formaldehyde and methanol in water) was used, as the exposure estimations do not explicitly account for variations in formalin formulation or expected volatilization from liquid form; although formalin is typically 37% formaldehyde, by weight, the range of methanol concentrations may vary from 6% to 12% (NIOSH, 2019). The possible exposure measurement errors and misclassifications are expected to be nondifferential, however, these types of errors can bias effects either toward or away from the null (Lash *et al.*, 2020). Therefore, accurate exposure estimation is critical for confidence in the identification of exposure-response relationships, and an accurate understanding of coexposures is necessary to identify or adjust for potential confounding or bias.

**Table 3.** Risk of bias ratings for epidemiological studies; supporting details provided in the supplemental materials

Study	Cohort	3 Appropriate comparison group	4*^a^* Confounding and modifying variables	7 Complete w/o attrition or exclusion	8*^a^* Exposure charac.	9*^a^* Outcome assessment	10 Selective reporting	Overall tier
Beane Freeman *et al.*, 2009	U.S. NCI formaldehyde	−	−	+	−	+	+	***2***
Checkoway *et al.*, 2015		−	−	+ +	−	+	+	***2***
Hauptmann *et al.*, 2009	U.S. funeral industry	+ +	+	+ +	−	+	+ +	***2***
Walrath and Fraumeni, 1984	Embalmers (NY, USA)	−	− −	−	− −	+	−	***2***
Walrath and Fraumeni, 1983	Embalmers (California, USA)	−	−	−	−	−	+	***3***
Stroup *et al.*, 1986	American Association of Anatomists	−	− −	−	− −	+	− −	***2***
Hayes *et al.*, 1990	U.S. embalmers and funeral directors	−	− −	+	− −	− −	+ +	***3***
Hall *et al.*, 1991	Royal College of Pathologists	−	− −	+ +	− −	+	−	***2***
Linos *et al.*, 1990	Embalmers and funeral directors	+	+	− −	−	+	+	***2***
Coggon *et al.*, 2014	British chemical workers	−	− −	+	− −	+	− −	***2***
Pira *et al.*, 2014	Laminated plastic factory workers	−	− −	+ +	− −	−	+ +	***3***
Meyers *et al.*, 2013	U.S. garment workers	−	−	+ +	−	+	+	***2***
Ott *et al.*, 1989	U.S. chemical manufacturing workers	−	−	+ +	− −	−	+	***3***
Andjelkovich *et al.*, 1995	Iron foundry workers	−	−	−	−	+	−	***2***
Gérin *et al.*, 1989	Cancer patients and pop. controls	−	+	−	−	+	+ +	***2***
Matanoski 1989	Pathologists	+	− −	−	− −	− −	+	***3***

Tier 1: A study must be rated as “definitely low” or “probably low” RoB for key elements AND have most other applicable items answered “definitely low” or “probably low” RoB.

Tier 2: Study meets neither the criteria for first or third tiers.

Tier 3: A study must be rated as “definitely high” or “probably high” RoB for key elements AND have most other applicable items answered “definitely high” or “probably high” RoB.

(++) = definitely low risk of bias; (+) = probably low risk of bias; (−) = probably high risk of bias; (− −) = definitely high risk of bias.

a Key question used for interpretation of quality of evidence and overall tier considerations.

The majority of studies also were considered “probably high” or “definitely high” risk of confounding bias, as most studies in the evidence base did not account for critical confounders such as smoking (a known risk factor for acute myeloid leukemia) and occupational coexposures such as the use of formalin or benzene. Only Hauptmann *et al.* (2009), Linos *et al.* (1990), and Gérin *et al.* (1989) accounted for smoking status, and only Gérin *et al.* (1989) accounted for all critical confounders. Gérin *et al.* (1989) did not identify a significant increase in Hodgkin lymphoma when comparing cases with population-based controls (OR: 0.5; 95% CI 0.2–1.4), however they did not evaluate other LHP subtypes of interest. Most studies adjusted for age, race, or sex, but failed to adequately account for other characteristics such as obesity. Adjustment for coexposures was limited; although some studies adjusted for potential coexposures to benzene or other occupational exposures (eg, Beane Freeman *et al.*, 2009), none accounted for coexposures to methanol through use of formalin. Lack of consideration or adjustment for these confounding or effect modifying factors may bias observed associations, usually away from the null.

RoB for outcome assessment was typically low, as most studies utilized ICD coding from death certificates to identify LHP cases; however only Gérin *et al.* (1989) evaluated LHP incidence instead of mortality, and so the total number of cases may be underreported.

Due to the likelihood of multiple types of systemic bias, none of the studies were considered Tier 1 (Table 3). The likely presence of residual and systemic bias related to exposure misclassification across these studies limits the ability to assess biological gradient and coherency within and across studies with respect to exposure-response. Exposure misclassification has less impact on the ability to assess consistency if it is assumed that all of the studies involved had increased (relative to non-occupational populations/controls) exposures to formaldehyde. In some cases, the use of occupational categorizations may be reasonable estimates that exposure occurred (though less reasonable regarding the actual exposure level), but in others—such as in the embalmer cohorts—uncertainties in not only the level but also the type and form of the exposure (eg, liquid solution versus inhalable formaldehyde), increase the potential impact of misclassifications and limit confidence in the observed associations.

Residual bias related to confounding cannot be ruled out; in particular, most of the studies did not consider the confounding impact of smoking, which is considered a significant limitation in reliability given that it is a known risk factor for several of the cancer types evaluated herein. These limitations in exposure and confounding collectively preclude confidence in reported associations from individual studies and also preclude certainty in strength, magnitude, and biological gradient across the evidence base. And, further, the RoB assessment demonstrates that it is possible that residual bias could explain or substantially contribute to the inconsistent findings observed in the few studies reporting associations.

#####  

Evidence was subsequently synthesized according to these outcomes; this approach is consistent with an understanding of the etiological diversity of LHP malignancies and NASEM (2011) recommendations. Of the available evidence, sufficient information was available to estimate pooled effect estimates for Hodgkin lymphoma, myeloid leukemia, and multiple myeloma with ever/never exposure to formaldehyde as measured by an SMR. Insufficient information was available for monocytic leukemias and lymphoid leukemia due to the limited number of publications and/or the limited number of publications with comparable effects estimates.

**Figure 2.**
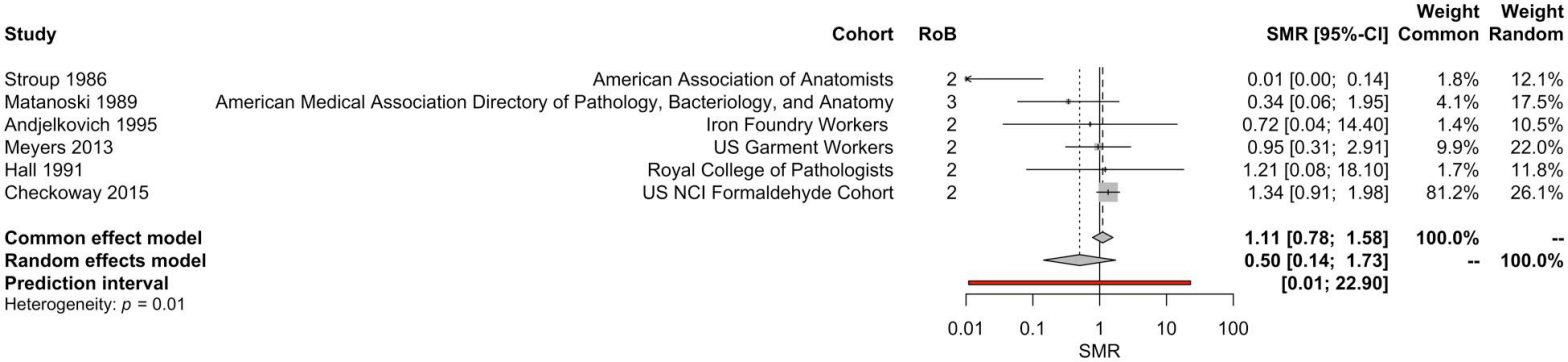
Meta-analysis of SMR estimates for Hodgkin lymphoma. Note—the 95% CIs for the SMRs reported in this figure differ slightly from those reported in the primary literature; these 95% CIs are calculated using the estimated SE of the lnSMR during the meta-analytic process. The SMR reported by Stroup *et al*. (1986) is 0, which was offset by 0.01 (see Materials and methods) for inclusion in this analysis.

Individual studies on Hodgkin lymphoma leukemia showed no evidence of a statistically significant association with ever/never exposure to formaldehyde (Table 2); meta-analyses did not identify a statistically significant association between inhaled formaldehyde exposure and risk of Hodgkin lymphoma (Figure 2 and Table 4).

**Table 4.** Summary of meta-analysis results (SMR for ever/never exposure in whole cohort)

Subtype	*N* studies	Fixed effects lnSMR (95% CI)	Random effects lnSMR (95% CI)	Egger test *p* value*^a^*	*I* ^2^; *p* value for heterogeneity
Hodgkin lymphoma	5	0.1056 (−0.247, 0.458)	−0.6917 (−1.93, 0.549)	.1368	66.6%; .01
Myeloid leukemia	4	0.0907 (−0.108, 0.289)	0.4127 (−0.335, 1.16)	.0443	76.5%; <.01
Multiple myeloma	4	0.0085 (−0.194, 0.211)		.8658	0.00%: .70

a Test for symmetry in funnel plot, an indicator of publication bias.

**Figure 3.**
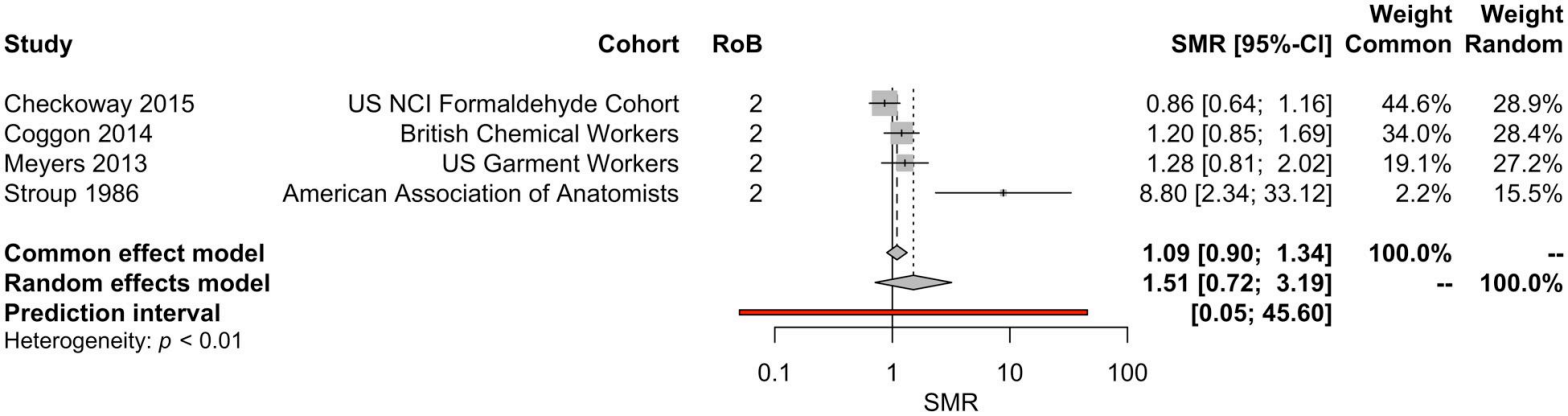
Meta-analysis of SMR estimates for myeloid leukemia. Note—the 95% CIs for the SMRs reported in this figure differ slightly from those reported in the primary literature; these 95% CIs are calculated using the estimated SE of the lnSMR during the meta-analytic process.

Studies evaluating associations with myeloid leukemia report mixed results; associations between mortality (measured as SMR) typically did not identify significant associations with formaldehyde exposure, with the exception of a significant increase in mortality reported by Stroup *et al.* (1986). However, Hauptmann *et al.* (2009) did identify a significant increase in the odds of myeloid leukemia mortality in a case-control analysis among embalmers. Meta-analyses did not identify a statistically significant association between inhaled formaldehyde exposure and risk of myeloid leukemia (Figure 3 and Table 4).

Significant between-study heterogeneity was identified in the meta-analyses of inhaled formaldehyde exposure and Hodgkin lymphoma and myeloid leukemia (*p* value for heterogeneity <.05 for each). This is largely due to the inclusion of observations from Stroup *et al.* (1986), the study of members of the American Association of Anatomists; the reported SMRs were substantially different compared with other studies included the analyses. *Post-hoc* sensitivity analyses show that removal of Stroup *et al.* (1986) from the meta-analyses reduces the RoB due to heterogeneity, but did not change the findings of the meta-analysis.

**Figure 4.**
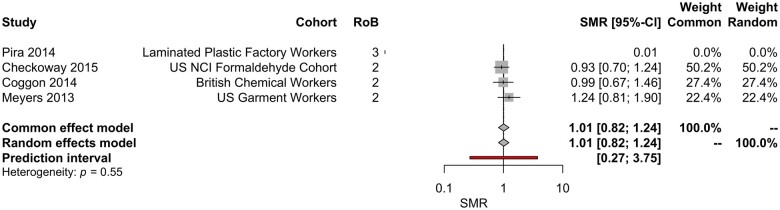
Meta-analysis of SMR estimates for multiple myeloma. Note—the 95% CIs for the SMRs reported in this figure differ slightly from those reported in the primary literature; these 95% CIs are calculated using the estimated SE of the lnSMR during the meta-analytic process. The SMR reported by Pira *et al*. (2014) is 0, which was offset by 0.01 (see Materials and methods) for inclusion in this analysis.

For multiple myeloma, no significant increases in SMRs were identified with ever/never exposure to formaldehyde, however, 1 study reported a statistically significantly increased PMR (Hayes *et al.*, 1990). Conversely, Pira *et al.* (2014) reported fewer than expected deaths from multiple myeloma in a cohort of plastic workers (0 observed deaths with 2.3 expected) and Checkoway *et al.* (2015) reported only a significant risk of multiple myeloma in the unexposed population. Meta-analyses did not identify a statistically significant association between inhaled formaldehyde exposure and risk of multiple myeloma (Figure 4 and Table 4).

Individual studies on lymphoid leukemia (Beane Freeman *et al.*, 2009; Checkoway *et al.*, 2015; Hayes *et al.*, 1990; Meyers *et al.*, 2013; Ott *et al.*, 1989; Walrath and Fraumeni, 1984) showed no evidence of a statistically significant association with ever/never exposure to formaldehyde (Table 2 and Supplementary Material 4).

Only 3 studies addressed monocytic leukemia, with mixed findings (Stroup *et al.*, 1986; Walrath and Fraumeni, 1983, 1984). Stroup *et al.* (1986) identified 1 death from acute monocytic leukemia, but did not report the expected number of deaths or an SMR or PMR; however, the authors imply that the findings were not significant, as they state that “the increased risk for leukemia was limited to the myeloid cell types.” Walrath and Fraumeni (1983, 1984) reported the total number of monocytic leukemia cases from cohorts of embalmers in New York and California, but did not calculate a PMR or SMR and did not evaluate statistical significance.

Additional sensitivity analyses did not indicate that the meta-analyses were sensitive to the inclusion or exclusion of lower-quality (Tier 3 RoB) studies, the selection of offset value for effects estimates based on 0 observed deaths, or the use of the SMRs reported by Beane Freeman *et al.* (2009) instead of those reported by Checkoway *et al.* (2015) for the NCI cohort (see Supplementary Material 9). Egger’s regression test indicated a potential presence of publication bias in the meta-analysis for myeloid leukemia (*p* = .04). Imputing unpublished studies using the Duval and Tweedie (2000) trim and fill method lowered the summary effect estimate for myeloid leukemia (lnSMR: 0.001; 95% CI: −0.91–0.91). Thus, publication bias is not expected to change the direction or significance of the findings.

Exposure-response was evaluated in some but not all studies; no consistent findings were observed across studies and appeared sporadic across metrics of cumulative, peak, average intensity, and duration of inhaled formaldehyde exposure. No statistically significant trends were identified with increasing categories of peak, cumulative, or average exposure and risk of lymphoid leukemia or multiple myeloma (Beane Freeman *et al.*, 2009; Checkoway *et al.*, 2015; Meyers *et al.*, 2013). Statistically significant trends were reported for Hodgkin lymphoma with increasing categories of peak exposure in both analyses of the NCI cohort (Beane Freeman *et al.*, 2009; Checkoway *et al.*, 2015), but the trend for increasing categories of cumulative exposure was only statistically significant in 1 analysis (Checkoway *et al.*, 2015), and the significance disappeared after excluding employees that worked <1 year. Among the studies evaluating myeloid leukemia, no statistically significant trends in the NCI, British Chemical Workers, or US Garment Workers cohorts for increasing peak, average, duration, or cumulative exposure (Beane Freeman *et al.*, 2009; Checkoway *et al.*, 2015). In the cohort of U.S. embalmers, statistically significant trends were reported with increasing duration of years worked in embalming and peak formaldehyde exposure, but not cumulative exposure or average intensity of exposure (Hauptmann *et al.*, 2009). Insufficient data were reported across studies to independently conduct meta-regression.

#### Assessment and synthesis of *in vivo* experimental animal evidence

There were 4 *in vivo* bioassays identified which met the inclusion and exclusion criteria of mammalian species. The study design was direct and generalizable to the PECO, as inhalation as the route of exposure, exposure to formaldehyde or formalin alone, and the purpose was to assess carcinogenicity, including in tissues outside the respiratory tract with potential relevance to LHP cancers (Albert *et al.*, 1982; Battelle, 1982; Kamata *et al.*, 1997; Morgan *et al.*, 2017; Sellakumar *et al.*, 1985). Data extracted from these studies focused on incidence of LHP cancers, hematological/clinical parameters, and specific organs related to LHP cancers (spleen, thymus, lymph nodes, bone marrow) (Table 5 and Supplementary Material 5).

**Table 5.** Summary of the identified experimental animal evidence investigating inhalation of formaldehyde and LHP tumor development; reliability categorization based on RoB and subject matter expertise (details provided in Supplemental Materials)

Citation	Study design	Results
Battelle, 1982	Rat (F344); male/female; 119–121/sex/group Whole-body exposure to 0, 2, 6, or 15 ppm formaldehyde (generated by heating paraformaldehyde) for 6 h/day, 5 day/week, 24 months Complete histopathology examination	Scattered statistically significant findings were observed in all study groups and each analysis period; due to the absence of trends it was determined these effects were not due to formaldehyde exposure. A significant change in the incidence of leukemia (all) was observed in female rats (see text).*^a^*
	Mouse (B6C3F1) male/female; 119–121/sex/group Whole-body exposure to 0, 2, 6, or 15 ppm formaldehyde (generated by heating paraformaldehyde); 6 h/day, 5 day/week, 24 months Complete histopathology examination	Scattered statistically significant findings were observed in all study groups and each analysis period; due to the absence of trends, it was decided these effects were not due to formaldehyde exposure. No significant changes were observed in organ weights (relative or absolute). Lesions in the thymus, spleen, lymph nodes, and bone were not significant. Incidence of leukemias (all) were not significant.
Kamata *et al.*, 1997	Rat (F344/DuCrj); male; 32/group Whole-body exposure to 0, 0.3, 2.0, 15 ppm formalin (a methanol control was also included); 6 h/day, 5 day/week, 28 months Major tissues and gross lesions were collected for histopathology	Macroscopic observation did not reveal any lesions other than nasal tumors. Similarly, no microscopic lesions attributable to formaldehyde exposure were detected in the organs other than the nasal cavity; however, histopathological examination of non-nasal tissues was limited. No changes were reported for hematological measures.
Sellakumar *et al.*, 1985; Albert *et al.*, 1982	Rat (Sprague Dawley); male; 100/group Whole-body exposure to 0 or 15 formaldehyde (sham control was also included); 6 h/day, 5 day/week, 128 weeks Complete necropsy with focus on respiratory tract	Metastatic lesions or tumors in organs as well as latency and mortality were not observed to be significantly different between treated and control groups. Three malignant lymphomas were reported; however, the incidence was not significant.
Morgan *et al.*, 2017	Mouse (B6.129-Trp53tm1Brd and C3B6.129F1-Trp53tm1Brd); male; 24–35/group Whole-body exposure to 0, 7.5, or 15 ppm; 6 h/day, 5 day/week, 8 weeks Major tissues and gross lesions were collected for histopathology at approximately 52 week of age	Differences in Spleen and thymus weights (relative and absolute), hematological parameters, leukemia, lymphoma, and neoplasms (lymphoma) in the lymph nodes and thymus were not significantly different from the untreated group. The neoplasms observed were determined to be unrelated to formaldehyde exposure.

a Increased bone marrow hyperplasia was significantly increased in male and female rats at the high dose (15 ppm).

#####  

RoB assessments were conducted for the animal studies (see Table 6 and Supplementary Material 8). Battelle (1982) was determined to be reliable and informative (internal and external validity, respectively); it was conducted and reported in a manner consistent with best practices at the time it was conducted, including a lengthy laboratory report with raw data. Although some RoB aspects of reporting were limited (eg, allocation information, characteristics of animals that were removed from the study, and necropsy results of animals found dead), these attributes were not considered major methodological limitations which would have substantially impacted the reliability of the study to assess exposure-response relationships. Generally, the design and execution was reliable, though it was recognized that the reporting of effects in the bone marrow were not extensively investigated given the focus on portal of entry tumors. Based on these considerations, Battelle (1982) was considered a Tier 2 study. Morgan *et al.* (2017) was considered reliable in regard to conduct, with some limitations in reporting. This study also had limitations in external and construct validity based on study design in regard to standard evaluations of carcinogenicity based on the 8-week exposure, though was purportedly designed to evaluate the potential role of the tumor suppressor p53 as related to LHPs (discussed further below). Therefore, based on these considerations, Morgan *et al.* (2017) was considered a Tier 1 study. Remaining studies were assigned to Tier 3 based on limitations in reporting on group allocation, purity of formaldehyde, blinding procedures, and not reporting all measured outcomes that died throughout the study.

**Table 6.** Risk of bias ratings for toxicological studies visualized as a heat map

Study	1 Dose or exposure level randomized	2 Study group allocation concealed	5*^a^* Experimental conditions identical	6 Research personnel blinded	7 Complete w/o attrition exclusion	8*^a^* Exposure charact.	9*^a^* Outcome assessment	10 All measured outcomes reported	Overall tier
Battelle, 1982	+ +	NR	+	+	−	+ +	+	−	***2***
Kamata *et al.*, 1997	−	NR	−	−	−	−	−	−	***3***
Sellakumar *et al.*, 1985	+	NR	+	NR	NR	−	−	+	***3***
Morgan *et al.*, 2017	+	NR	+	NR	+	+	+	+ +	***1***

Tier 1: A study must be rated as “definitely low” or “probably low” RoB for key elements AND have most other applicable items answered “definitely low” or “probably low” RoB.

Tier 2: Study meets neither the criteria for first or third tiers.

Tier 3: A study must be rated as “definitely high” or “probably high” RoB for key elements AND have most other applicable items answered “definitely high” or “probably high” RoB.

(++) = definitely low risk of bias; (+) = probably low risk of bias; (−) = probably high risk of bias; (− −) = definitely high risk of bias; NR = not reported.

a Key question used for interpretation of quality of evidence and overall tier considerations.

#####  

Because formaldehyde is a reactive gas, most inhalation studies have primarily focused on portal of entry effects in the nasal cavity and respiratory tract. Data also indicate that inhaled formaldehyde does not distribute systemically beyond the portal of entry (see biological plausibility below). As such, there has been comparatively less focus on toxicity in systemic organs; however, 4 studies are available to inform LHP—2 lifetime studies (Albert *et al.*, 1982; Battelle, 1982; Sellakumar *et al.*, 1985); and 2 studies with study design specific to assessing carcinogenic properties (Kamata *et al.*, 1997; Morgan *et al.*, 2017).

Battelle (1982) was the only 2-year bioassay that fully assessed LHPs following exposure to inhaled formaldehyde in both mice and rats for 24 months (a standard and well-accepted timeframe to represent lifetime exposure). Animals (120 per dose group) were exposed to 0, 2, 6, or 15 ppm for 24 months; interim necropsies were conducted at 6, 12, and 18 months. A significant increase in diffuse, multifocal hyperplasia was observed in the bone marrow of male and female rat femurs; study authors indicated that these lesions may be associated with an increased demand for leukocytes and erythrocytes—the direction of this finding is opposite of what would be expected for an agent that has effects similar to those of other known myeloleukaemogens (IARC, 2012). The occurrence of all types of leukemia was found to be significantly different in the 15 ppm group compared to the control group in female rats when examined using a pairwise adjusted Cox/Tarone test (*p* = .0056), but not an unadjusted exact test (*p* = .2316). There is some ambiguity on the direction of this significant difference as the unadjusted incidence in the 15 ppm group (6%) is lower than the control group (9%). The adjusted *p* value accounts for time to the tumor and thus the significance of the adjusted value might be indicative of an increase; however, USEPA (2022) clearly stated that the incidence of leukemia in female rats is significantly decreased. These results collectively support a lack of LHPs following high doses of exposure to formaldehyde for a lifetime in both rats and mice.

Albert *et al.* (1982) and Sellakumar *et al.* (1985) provide results from a study involving a single concentration of formaldehyde (15 ppm) over a lifetime in rats. Authors report that no significant increase in tumors was observed beyond the nasal cavity at necropsy. Kamata *et al.* (1997) exposed rats to 0, 0.3, 2, and 15 ppm formaldehyde formalin for 28 months. At study termination, no significant effects were observed in the lymph nodes or femur, and no significant changes in lymphocyte counts were observed (Kamata *et al.*, 1997).

Morgan *et al.* (2017) was conducted by the National Toxicology Program (NTP); it was specifically focused on evaluating the potential role of the tumor suppressor p53 and thus was conducted in genetically engineered mice heterozygous for Trp53. (It should be noted that an earlier publication of this NTP report described the purpose of the study differently [Morgan *et al.*, 2015]: “Formaldehyde (FA) inhalation is linked to myeloid leukemia in humans, although the mechanism is unclear. DNA adduct formation is recognized as a key mechanistic event in FA-induced nasal cancer; however, inhaled FA does not form detectable levels of DNA adducts at sites other than the nasal cavity. It was hypothesized that FA may cause leukemia by a mechanism not involving DNA adduct formation. Inhaled FA could cause significant genetic damage to stem cells in the nasal epithelium or circulating in local blood vessels. These damaged stem cells could reach the general circulation, home to tissues that support the hematopoietic niche, undergo lodgment and become leukemic stem cells. We tested this hypothesis…Under the conditions of this study, FA inhalation did not cause leukemia or lymphohematopoietic neoplasia in these genetically predisposed mice.”) Two strains of haploinsufficient Trp53 mice were exposed to 0, 7.5, or 15 ppm formaldehyde for 8 weeks, monitored for an additional 32 weeks, and necropsied at approximately 52 weeks of age. The inbred B6.129-*Trp*53^tm1Brd^ mouse strain was selected because it has been previously used as a model for LHP tumors in short-term cancer bioassays (Morgan *et al.*, 2017). The second strain, C3B6.129F1-*Trp*53^tm1Brd^, has been reported to display treatment-related LHP tumors induced by other chemical compounds (Eastmond *et al.*, 2013; Morgan *et al.*, 2017). These models are also reported to be ideal for detecting genotoxic carcinogens (Eastmond *et al.*, 2013). Similar to chronic bioassays, no significant changes were observed in the incidence of LHPs, relevant organ weights (spleen and thymus), or hematological parameters.

In summary, all available studies consistently report a lack of association or occurrence of LHPs. It is recognized that these studies may have limitations related to the general focus on respiratory tissues and gross lesions of reported histopathological examinations. However, Battelle (1982) comprehensively evaluated tissues associated with LHP (USEPA, 2022), and thus the lack of occurrence of LHPs in this study provides strong experimental evidence demonstrating a lack of causal relationship of inhaled formaldehyde and LHP cancers.

#### Evidence integration and assessment of causal relationship

Consistent with recommendations from GRADE and OHAT, the epidemiological evidence are generally associated with low to moderate confidence, owing primarily to the lack of controlled exposure and study design; experimental animal data, in contrast, have an initial high level of confidence due to controlled exposure and study design (Table 7). Given the inconsistent and mixed findings reported in human studies, evidence was assessed both in context of the presence of a causal relationship as well evidence demonstrating lack of a causal relationship.

**Table 7.** Application of Bradford Hill considerations and OHAT confidence ratings for integration of evidence across epidemiology, toxicology, and mode of action.

Criterion	Evidence stream	
	Human	Experimental animal
**Initial synthesis of evidence**	Taken together, the majority of human evidence reports a lack of association between formaldehyde inhalation and LHPs across occupational cohorts. Positive findings are limited to inconsistent reports of increased mortality from myeloid leukemia, monocytic leukemia, or multiple myeloma (typically when associations are measured as a PMR) or exposure-response trends in Hodgkin lymphoma or myeloid leukemia, as reported in individual studies. None of these cancers were elevated in pooled analyses.	Individual studies all report that formaldehyde exposure does not lead to a significant increase in the incidence of LHP-related lesions.
**Initial confidence rating**	Low-to-moderate (++ to +++) confidence	High (++++) confidence
RoB; residual and confounding bias	Decrease confidence due to high RoB within and across evidence stream. No studies involved exposure measurements, most studies did not account for key confounding (eg, smoking), co-exposures, and effect-modifying variables. Decreased confidence in causal relationship given likely impact of confounding bias related to smoking and benzene exposures).	Slight decrease in confidence due to the RoB across studies. However, the biases are not expected to impact the ability to assess treatment-related effects or, subsequently, causality. Experimental studies generally conducted according to best-laboratory practices at the time of conduct.
Consistency/unexplained inconsistency	Decrease in confidence in the presence of causal relationships due to inconsistent findings across studies and within studies (by metric) and tumor types; most individual studies and meta-analyses report a lack of association. Inconsistent findings of selected individual studies are potentially explained by residual bias related to confounding and exposure misclassification.	Increase confidence in lack of causal relationship: evidence in rats and mice at similar dose groups and similar exposure scenarios to formaldehyde or formalin is consistent.
Directness	No increase or decrease: though data are limited to occupational exposures in men, evidence was still considered generalizable to the assessment of causation in humans.	Slight decrease in confidence: standard animal bioassays designed to evaluate the outcome were utilized and directly inform the assessment of causation and treatment-related effects; includes assays unique to assessing species specificity. Some limitations given that most bioassays conducted were focused on portal of entry tissues, and the need to extrapolate to humans.
Publication bias	No increase or decrease—publication bias was detected for assessments of myeloid leukemia, but the impact is not expected to change the direction or significance of the meta-analysis findings.	Not assessed.
Strength of association (magnitude)	Decreased confidence in the presence of causal relationship: When associations were observed, the magnitude was low. The magnitude of reported SMR estimates for ever/never exposures is generally a ratio of <1.5 for multiple myeloma, Hodgkin lymphoma, and myeloid leukemia, however there is a large amount of uncertainty and variability, and so these effect estimates are generally not statistically significantly increased; meta-analyses of ever/never SMR estimates indicate there is no significant association between formaldehyde inhalation and mortality from Hodgkin lymphoma, multiple myeloma, or myeloid leukemia. Some ORs and HRs for highly exposed workers have a greater magnitude of effect, but less precision, and many are not statistically significant.	Increased confidence in lack of causal relationship; no treatment-related LHPs observed.
Imprecision	No increase or decrease in confidence in causal relationship—some identified studies are limited in population size and selection, which imparts high uncertainty in effect estimates. However, large studies with precise effects estimates were identified (eg, NCI cohort; Beane Freeman *et al.* 2009; Checkoway *et al.*, 2015). Use of meta-analysis increases sample size to account for imprecision in individual studies.	Increased confidence in lack of causal relationship—studies monitored formaldehyde levels and the measured exposures and responses are considered sufficiently precise.
Exposure-response gradient	Decreased confidence in causal relationship—when associations were observed, exposure-response gradients are not consistently observed. No statistically significant trends were identified with increasing categories of peak, cumulative, or average exposure and risk of lymphoid leukemia or multiple myeloma (Beane Freeman *et al.*, 2009; Checkoway *et al.*, 2015; Meyers *et al.*, 2013). However, Hauptmann *et al.* (2009) reports an exposure-response gradient for myeloid leukemia based on cumulative exposure among embalmers. Beane Freeman *et al.* (2009) and Checkoway *et al.* (2015) identify a gradient for Hodgkin lymphoma based on peak and cumulative exposures in the NCI cohort.	Increased confidence in lack of causal relationship—consistent reports of a lack of dose-response.
Specificity	No change in confidence—LHPs are not specific to formaldehyde exposures, and may be caused by alternative exposures (including, but not limited to benzene, ionizing radiation, and smoking).	No change in confidence—other chemicals, such as benzene, are known to cause LHPs.
*Final rating (confidence in evidence)*	***Very low (+) confidence in body of evidence***	***High (++++) confidence in the body of evidence***
**Additional considerations for causation (not assessed in OHAT and GRADE)**		
Temporality	Increased confidence in lack of causal relationship; exposure preceded response.	
Biological plausibility	Increases confidence in lack of causal relationship. Evidence consistently demonstrates that inhaled formaldehyde does not distribute beyond the portal of entry. Evidence consistently shows that inhaled formaldehyde is not linked to increases in blood formaldehyde levels.	
Experiment	Increase confidence in lack of causal relationship—no evidence of treatment-related effect in experimental studies.	
Coherence	Decreased confidence in a causal relationship due to lack of coherence within and across evidence streams; most evidence support lack of causal relationship. The low magnitude increases in LHP cancer risk reported in highly exposure workers from some studies is not reproduced across populations or exposure metric. Effect is not reproduced in experimental animal studies utilizing doses much higher than those experienced in the occupational cohorts.	
Analogy	No change in confidence—lack of conclusive evidence that other aldehydes (eg, acetaldehyde and acrolein) are leukemogenic. IARC does not include any other aldehyde or acrolein in their lists of carcinogenic agents with *sufficient evidence* or *limited evidence* for LHP cancers (IARC, 2023).	
Final conclusion on causality	Available evidence does not support a causal relationship between inhalation of formaldehyde and development of LHP cancers. The inconsistent and small-magnitude associations reported between formaldehyde exposures (estimated, not measured) in some observational studies do not provide sufficient evidence of a causal relationship given the likelihood of residual bias and concurrent consideration for the lack of consistency and coherency with other observational data, experimental evidence, and lack of biologically plausible explanation—particularly given that data from these lines of evidence consistently support a lack of causal relationship.	

#####  

RoB was substantial across all observational data; though some Tier 2 human studies were identified, the limitations in such (eg, no measured exposure data, likely presence of confounding due to smoking or co-exposures) were considered to have more significant impact on the ability to assess a true association between exposure and outcome than those limitations in the experimental animal data whereby exposure was measured, confounding was not present, and outcome was directly measured. For example, the experimental animal evidence from Battelle (1982) wherein only the high dose and control were comprehensively evaluated histopathologically (tissues from other doses were not assessed unless gross lesions were observed—a standard practice) were considered at less risk of systemic bias than the observational studies using ever/never categorizations with uncontrolled confounding due to smoking and other variables.

###### Consistency, directness, magnitude, precision, publication bias, exposure-response, specificity, experiment, analogy, and coherence

Consistency, directness, magnitude, precision, exposure-response, and other evidence integration considerations are described in Table 7. As the majority of evidence in the toxicological stream either increased or had no change on confidence, this section focuses on the evidence reporting associations in the human evidence stream. Confidence was decreased based on the likelihood of confounding and residual biases, the relatively low magnitude of effect, and the lack of exposure-response in the epidemiological evidence. These attributes apply by cancer type and by exposure metric. Use of meta-analyses increased the sample size and improved precision relative to individual studies; as such, there is more confidence in the meta-analytical findings due to a higher level of precision.

###### Biological plausibility: assessment based on endogenous formation, toxicokinetics, mode of action, and genotoxicity

Much research has been conducted which informs the assessment of the biological plausibility of formaldehyde exposure and cancer. This includes assessment of inhalation dosimetry as it relates to genotoxicity and mode of action. In the body of literature, the focus has largely been placed assessing the potential human relevance of portal of entry tumors observed in rodents; however, this research also informs the assessment of plausibility of non-portal of entry tumors (and specifically LHPs) in humans. Additionally, investigations have been conducted to directly assess biological mechanisms underpinning plausibility of leukemia. These lines of evidence have been subject to large research efforts each on their own and are summarized herein in context of assessing biological plausibility of a causal relationship between formaldehyde inhalation and LHPs.

###### Endogenous formaldehyde

Formaldehyde is naturally present endogenously in all tissues. It is generated in both the cytoplasm and the nucleus as part of normal cellular processes. Sources of endogenous formaldehyde include N-, O-, and S-demethylation reactions, the one-carbon pool (or 1C cycle), and DNA demethylation reactions (Heck and Casanova, 2004; Walport *et al.*, 2016). Natural exogenous sources include foods, where both formaldehyde and methanol (which is metabolized to formaldehyde) are naturally present (IARC, 2006). Enzymatic detoxification of formaldehyde is primarily mediated by 2 pathways: one involving glutathione-dependent formaldehyde dehydrogenase, also called class-III alcohol dehydrogenase (ADH3) in rodents and ADH5 in humans (Koivusalo *et al.*, 1989), and the other is aldehyde dehydrogenase-2 (ALDH2). In the former pathway, formaldehyde first reacts with GSH to form hydroxymethyl glutathione (HMGSH) that is reduced by ADH3/5 to S-formylglutathione (FGSH), and this product is then hydrolyzed by S-formylglutathione hydrolase (FGH) to formate along with regeneration of GSH (Uotila and Koivusalo, 1974). ALDH2 metabolism is likely relevant under higher exposure conditions due to the overall low affinity of ALDH2 for formaldehyde (Staab *et al.*, 2008; Teng *et al.*, 2001). Nonenzymatic reactions of free formaldehyde occur via reversible binding with cellular macromolecules such as proteins and DNA; entry into the 1C cycle leads to irreversible incorporation into macromolecules (Burgos-Barragan *et al.*, 2017).

###### Toxicokinetics: inhalation dosimetry

There is a significant body of literature spanning decades and involving increasingly sophisticated and sensitive methods demonstrating that inhaled formaldehyde does not distribute beyond the portal of entry. For example, studies in rats and monkeys reported that blood formaldehyde levels were unchanged following high-dose exposures (Heck *et al.*, 1985; Kleinnijenhuis *et al.*, 2013). Exposure of F344 rats to 10 ppm [^13^CD_2_]-formaldehyde for 6 h/day for 1 or 5 days resulted in exogenous formaldehyde-DNA adduct formation in nasal tissue but not in the bone marrow or spleen (Alliance for Risk Assessment, 2022; Lu *et al.*, 2010). Similarly, exposure of cynomolgus macaques to 6 ppm [^13^CD_2_]-formaldehyde for 6 h/day for 2 days resulted in exogenous formaldehyde-DNA adduct formation in nasal tissue but not in the bone marrow (Moeller *et al.*, 2011). Consistent with these studies in laboratory animals, humans exposed to 1.9 ppm unlabeled formaldehyde for 40 min did not exhibit significant increases in blood formaldehyde (Heck *et al.*, 1985). Studies in transgenic animal models deficient in formaldehyde metabolism have shown that large increases in serum formaldehyde are required to increase endogenous formaldehyde-DNA adduct levels compared with wild-type mice (Dingler *et al.*, 2020), indicating that extremely large increases in serum formaldehyde (ie, those sufficient to overwhelm metabolism of exogenous formaldehyde) would be needed to elicit systemic effects. Collectively, these studies preclude the possibility for inhaled formaldehyde to directly increase DNA damage beyond the portal of entry, thus supporting a lack of a biologically plausible pathway for formaldehyde to cause LHP cancers via a genotoxic mode of action.

###### Genotoxicity

The genotoxicity of formaldehyde has been recently reviewed elsewhere (Thompson *et al.*, 2020). Formaldehyde is unequivocally genotoxic *in vitro*. In bacterial assays, formaldehyde induces point mutations, insertions and deletions (IARC, 2006). In mammalian cells, formaldehyde increases DNA-protein crosslinks, clastogenicity (micronuclei; MN), and cytotoxicity all within a similar range of concentration with limited evidence for gene mutation or aneugenic mechanisms (Albertini and Kaden, 2017; Merk and Speit, 1998; Speit *et al.*, 2011). There is little evidence for *in vivo* genotoxicity in controlled animal and human experiments (Thompson *et al.*, 2020). Despite such, the most frequently cited mechanistic support for formaldehyde to induce leukemia comes from observational studies in occupational cohorts that report findings inconsistent with the myriad of controlled exposure studies in rodents and humans.

A recent systematic review by Fenech *et al.* (2016) reviewing 17 studies reporting micronucleus results using the cytokinesis block micronucleus assay in lymphocytes (L-CBMN) from workers exposed to formaldehyde reported a statistically significant doubling of micronuclei in workers from resin and plyboard industries as well as pathology or research settings. Importantly, the authors noted several limitations to their analysis including: (1) the majority of the occupational exposure studies only reported average air concentrations, (2) there was a high level of heterogeneity between investigations in terms of a number of subjects in the control and exposed groups, the duration of exposure, the exposure setting (pathology laboratories, plywood manufacture, controlled exposures in laboratory), the design of the studies (ie, cross-sectional control group/exposed group vs before/after exposure in the same subject); and (3) exposure to other genotoxic chemicals that might have been present together with formaldehyde in the occupational setting was not reported or not reported quantitatively. The authors also noted that meta-analysis was not conducted because of the high heterogeneity between studies. Similar limitations have been noted in other reviews of these studies (Gentry *et al.*, 2013; Mundt *et al.*, 2017), and suggest that the inconsistent findings in the observational studies relative to the controlled exposure studies could be spurious and/or could be explained by residual biases.

###### Mode of action

Despite the evidence against systemic delivery, a few mechanisms for how formaldehyde might cause LHP cancers have been hypothesized without the formal application of MOA frameworks (eg, Zhang *et al.*, 2010, 2013). Gentry *et al.* (2020) recently compiled and reviewed 4 proposed mechanisms by which inhaled formaldehyde might cause leukemia: (1) initiation of leukemia by direct damage to hematopoietic stem cells in the bone marrow, (2) toxicity to circulating blood stem cells and progenitors, (3) direct damage to pluripotent stem cells of the nasal or oral cavity, and (4) targeting of blood stem cells and progenitors within the lung. These mechanisms were organized into MOAs so that key events could be evaluated using Bradford-Hill criteria. The authors concluded that the available supporting evidence for the key events in these postulated MOAs did not provide evidence of dose-response, temporal association, or biological plausibility between the key events. Notably, the first 2 MOAs require systemic delivery to blood or tissues. As discussed above, there is no evidence of systemic delivery to the bone marrow or blood following inhalation exposure to formaldehyde. Although it is theoretically conceivable that a blood stem cell passing through the nasal cavity might be exposed to formaldehyde, there are no dosimetry data supporting this hypothesis. Furthermore, inhalation genotoxicity studies in rodents exposed up to 15 ppm formaldehyde up to 8 weeks did not exhibit DNA damage in blood or bone marrow cells (Dallas *et al.*, 1992; Speit *et al.*, 2009). Human volunteers exposed to up to 0.7 ppm for 4 h/day for 5 consecutive days did not exhibit DNA damage in peripheral blood cells (Zeller *et al.*, 2011).

In contrast to the blood cells, labeled formaldehyde studies indicate that formaldehyde does interact with DNA at the portal of entry. Although adducts and DNA-protein crosslinks have been observed, as reviewed in Thompson *et al.* (2020), evidence of clastogenic and mutagenic damage are lacking (Meng *et al.*, 2010; Speit *et al.*, 2007, 2011; Zeller *et al.*, 2011). These results suggest that direct DNA damage to pluripotent cells of the nasal tissue or lung appears unlikely and has not been demonstrated. As discussed in Thompson *et al.* (2020) and in McGregor *et al.* (2006), cytotoxicity-induced regenerative hyperplasia is the likely driver of carcinogenicity in the rodent nasal cavity, with perhaps exceedance of endogenous formaldehyde-DNA levels in the nasal tissue following exposure to ≥15 ppm. According to Gentry *et al.* (2020), the hypothesis that nasal stem cells at the portal of entry might migrate to the bone marrow is based on a study by Murrell *et al.* (2005) that showed that rat olfactory stem cells could be injected into irradiated rats and give rise to hematopoietic lineages. Similarly, a single study was identified that supported the possibility that hematopoietic stem cells in the lung could recirculate back to the bone marrow (Lefrançais *et al.*, 2017). Neither this study nor Murrell *et al.* (2005) involved inhalation exposure to formaldehyde or any other agent.

###### Conclusions on biological plausibility

The ability of a substance to reach a target tissue is a fundamental concept to assessing the plausibility of an exposure to cause a response; a substantial body of evidence spanning decades and involving increasingly sophisticated and sensitive methods demonstrates that inhaled formaldehyde does not distribute beyond the portal of entry. The reported changes in genotoxicity markers in observational worker studies is inconsistent both with the evidence against systemic distribution as well as the results of controlled exposure studies directly assessing genotoxicity. As reviewed in Thompson *et al.* (2020), nearly all *in vivo* genotoxicity studies conducted with controlled formaldehyde exposure are negative, including in human buccal, nasal, and peripheral blood cells and nasal tissues of rats exposed up to 15 ppm formaldehyde for 4 weeks. Moreover, animal studies suggest that large increases in serum formaldehyde (substantially higher than endogenous levels) are necessary to cause DNA damage (Dingler *et al.*, 2020).

Formaldehyde is rapidly metabolized and therefore does not bioaccumulate. The average lifespan of a human lymphocyte is approximately 200 days, and therefore might accumulate chemicals or DNA damage (eg, oxidative damage) that manifests as micronuclei when stimulated to proliferate in the *ex vivo* L-CBMN assay. The likelihood that this is a result of formaldehyde exposure is highly uncertain. When recognizing the uncertainties in the observational data (eg, lack of certainty in exposure, confounding exposures, etc.,) relative to the consistent negative findings in controlled exposure studies, more weight is placed on findings from the controlled exposure studies with regards to informing biological plausibility herein. These data, combined with consideration of toxicokinetics, support that genotoxicity is not a plausible mode of action for development of LHP following inhalation of formaldehyde in humans.

## Discussion

The currently available evidence, as identified and evaluated in this systematic review, do not support that inhaled formaldehyde is causally related to LHP cancers, including Hodgkin lymphoma, multiple myeloma, myeloid leukemia, monocytic leukemia, or lymphoid leukemia. Meta-analyses of human studies and experimental animal studies consistently demonstrate a lack of association between formaldehyde exposure and LHP. The use of formal critical appraisal methods via RoB—a standard systematic review component which has not yet been applied to this evidence base—allowed for transparent and objective identification of attributes important to assessing causal relationships. This assessment demonstrated attributes a high likelihood of systemic and residual biases; these threats to internal validity were considered substantial with respect to assessing causality, and in particular, the likely role of confounding bias related to smoking and coexposures which could reasonably explain the inconsistent findings, particularly when combined with other causal criteria including biological plausibility, experiment, coherence, and biological gradient.

It is difficult to directly compare the findings of this assessment to other reviews, particularly authoritative reviews by IARC (2006, 2012), USEPA (2022), and other published reviews, given the difference in objectives and methodology: (1) the general objective involving evaluation of causation versus hazard characterizations, (2) the use of formal critical appraisal tools, and (3) the implementation of meta-analyses of LHP cancer mortality (reported as SMR). Existing guidance on the use of systematic review to facilitate hazard and risk assessment from the NTP, IARC, and WHO are not specific to the evaluation of causation. It is recognized that these agencies are conducting assessments for specific purposes and under such, may not (or do not) require that causation be established to determine potential hazards. Thus, it is reasonable that when looking at the same evidence base for a different purpose, the results appear in conflict. Herein, a systematic and focused assessment of the causal relationship between inhaled formaldehyde and LHP was conducted, which integrated critical appraisal of epidemiological data, meta-analyses of epidemiological data, experimental animal evidence, and careful examination of biological plausibility, thus differentiating the objectives and underlying evidence bases from existing reviews.

The meta-analyses conducted herein address previous limitations in other meta-analyses (Bachand *et al.*, 2010; Bosetti *et al.*, 2008; Collins and Lineker, 2004; Schwilk *et al.*, 2010; Zhang *et al.*, 2009) as related to the assessment of causality: (1) pooled estimates by LHP subtype (vs inappropriate combination of LHP subtypes into a single pooled estimate), (2) reduce redundancies in pooled effect estimates due to inclusion of workers from the same cohorts, (3) application of a systematic review method to ensure transparently defining study inclusion *a priori*, and (4) formal critical appraisal to assess the potential RoB. The results of this meta-analysis differ, particularly for the pooled effects reported for myeloid leukemia and multiple myeloma, from those reported by Zhang *et al.* (2009) and Schwilk *et al.* (2010). This is due, in part, to differences in included and excluded evidence which, for this analysis, were supported by the use of transparent systematic methods and use of only the most recent updates for each cohort. Additionally, this current analysis benefits from the use of updated information for multiple cohorts (including the NCI cohort, the U.S. garment workers, and British chemical workers) that were published after the completion of previously published analyses.

The pooled effect estimates measured through meta-analysis are limited in utility and interpretation due to the RoB in the underlying evidence base. Additionally, some evidence is lost due to the inability to appropriately combine SMRs with ORs or other proportional mortality effect estimates, and the inability to adequately account for highly exposed workers in pooled analyses. Individual inclusion of subsets of workers with the highest peak or cumulative exposures within a cohort may increase the pooled effects estimates. However, the effect estimates based on relative exposure estimates for workers in the NCI cohort (Beane Freeman *et al.*, 2009; Checkoway *et al.*, 2015) and embalmers (Hauptmann *et al.*, 2009) report mortality as an odds ratio, hazard ratio or relative risk, which is not appropriate for combination with SMRs. Additionally, combination of studies with derived RRs based on different exposure metrics (ie, peak, cumulative, and duration) and ranges is also not feasible, as the reported exposure ranges vary greatly between cohorts and studies.

A meta-regression that incorporates considerations of exposure, including intensity and frequency, population characteristics, coexposures, and other critical explanatory variables would be an ideal tool for addressing the observed heterogeneity among studies. However, the currently available evidence is not sufficient for meta-regression to support dose-response analysis due limited reporting and high RoB in exposure characterization (Table 3). These limitations preclude confidence in any analysis based on exposure categorization or measurement due to high levels of uncertainty in the estimated exposure concentrations. Additionally, uncertainty remains regarding the importance of peak or cumulative exposures on LHP tumor formation, as there is inconsistency in the epidemiological evidence and a lack of clear exposure-response trends across studies. Current guidance recommends reliance upon cumulative exposure estimations in the absence of clear mechanisms (NASEM, 2023). However, the exposure-response gradient reported for the NCI cohort is only consistently statistically significant when formaldehyde peaks are measured, not cumulative exposure (Beane Freeman *et al.*, 2009; Checkoway *et al.*, 2015). Although Checkoway *et al.* (2015) identified a significant exposure-response trend with cumulative formaldehyde exposures and Hodgkin lymphoma in the total NCI cohort (*p* = .02), those findings were attenuated when the analyses were adjusted to include only workers with ≥1 year of occupational exposure (*p* = .05), which is a reasonable adjustment to account for uncertainties in worker exposures and healthy worker bias. Ultimately, there is no clear exposure-response relationship from which a pooled gradient could be determined.

Some concerns have been raised that the most recent updates of the formaldehyde-exposed cohorts may underestimate risk, compared with their prior reported findings, due to a comparatively short induction and latency period for other leukemogenic chemicals (NIEHS, 2022). The magnitude of estimated effects estimates has decreased over time for some cohorts (eg, garment workers; see Pinkerton *et al.* [2004] compared to Meyers *et al.* [2013]). However, the SMRs are calculated based on the expected number of mortality events, based on the general population, and therefore should be reflective of true risk. Many of the studied epidemiological cohorts reported that the best fit for the risk models when lagging exposure by 15+ years to account for latency (Beane Freeman *et al.*, 2009), although information regarding induction and latency periods for LHPs is uncertain, and largely depending on the LHP type (eg, lymphoma vs leukemia, or chronic vs acute) (World Trade Center Health Program, 2014). Other studies have indicated that time since first exposure is predictive of LHP risk (Hauptmann *et al.*, 2009). Use of the most recent cohorts for the meta-analyses is consistent with these findings. Ultimately, findings of exposure-response associations based on duration of exposure are not consistent with the toxicological evidence (in which high, lifetime exposures to formaldehyde do not induce LHP cancers). Conversely, an underreporting of risk could be attributed to the use of LHP mortality outcomes instead of incidence for calculating risk. As reported in the NTP RoC (2021), studies reporting mortality for cancers with higher survival rates (such as LHP cancers), are less informative than studies reporting incidence, as mortality measures will miss cases that do not result in death. However, the lack of plausible MoA for systemic distribution of exogenous formaldehyde and lack of tumor development in the toxicological evidence indicate that underreporting in the epidemiological evidence is not of concern. In order to increase confidence in the observed associations from the epidemiological literature, further research is needed to identify additional biologically plausible mechanisms by which inhaled formaldehyde could induce LHP malignancies.

Use of systematic review methodology to evaluate the association between formaldehyde inhalation and potential LHP outcomes offers a robust and rigorous approach to evaluating and summarizing scientific evidence from multiple data streams. It is recognized, however, that systematic review methods are still evolving for use in risk assessment. This is highlighted by critical appraisal tools applied herein; RoB tools are highly applicable to epidemiological studies given their origin in medicine and nutrition and readily allowed for assessment of systemic bias. However, these same concepts are more often regarded as not sufficiently addressing all aspects of reliability and relevance of individual studies (eg, aspects beyond internal validity that are important on an individual study basis). This is highlighted in the evaluation of the study by Battelle (1982)—a robust cancer bioassay conducted in multiple species with an overall low level of RoB related to internal validity, but potential limitations regarding construct validity given the general focus on portal of entry tumors. USEPA (2022) also described having “high confidence” in this study with regards to methods, conduct, and reporting, yet characterized evidence as “indeterminate” for experimental animal data primarily because this histopathology was not assessed at all doses but rather focused on the control and high doses with respect to LHP tumors (a standard approach in toxicology when no findings are observed at the high dose). This demonstrates a lack of pragmatism when using systematic review in risk assessment when observational studies with associations based on “ever/never” estimates of exposure (ie, nonmeasured exposures) that may be impacted by coexposures, confounding, or other risk factors are weighted higher than controlled exposure studies designed specifically to evaluate cancer; animal studies are able to eliminate covariates of concern and provide accurate quantitative estimates of exposure and test for or establish an exposure-response gradient.

Few assessments and reviews have considered human and experimental streams in the evaluation of potential causality. Most recently, the USEPA (2022) considered several aspects of causation, including discussion on plausibility. However, in discussing the findings of associations from a subset of observational studies, the Agency did not fully incorporate experimental evidence or modern causation assessment techniques to better inform conclusions and uncertainties. The USEPA (2022) concluded that there was a causal role in LHP cancer development despite acknowledgment of the availability a robust cancer bioassay in 2 species combined, consideration that formaldehyde is unlikely to move or distribute beyond the portal of entry, and recognition that there is no known MOA for how formaldehyde might cause leukemia. Biological observations, rather than MoA, were used to support their conclusions based on observational studies in worker cohorts. As such, it is paradoxical that EPA made such conclusions regarding animal data and biological plausibility without attempting to characterize systemic bias, despite repeated recommendations from NASEM to do so (NASEM, 2011, 2023). The USEPA also failed to assess the impact of systemic biases and uncertainties in the subset of observational data reporting associations. These considerations should be accounted for through the application of critical appraisal and formal integration of evidence. For these reasons, the conclusions of this systematic review (which applied critical appraisal, evidence integration and RoB assessment) differ from those of the USEPA. Practically, this shows the critical need for systematic evidence integration and considerations of biological plausibility to improve confidence in hazard characterization, selection of critical endpoints and subsequent development of toxicity values or occupational exposure limits. Based on the findings of this review, LHP cancers do not appear to be suitable for use as a critical effect in FA risk assessments.

The RoB presented in this analysis demonstrates a likelihood of systemic bias which precludes confidence in drawing conclusions from the observational data. To improve confidence in using these studies, identifying the direction and quantitative impact of residual bias would better inform causality and risk assessment decisions. The RoB analysis has identified attributes which should be further explored using quantitative modern causal inference and bias-adjustment methods. Some quantitative methods, such as Bayesian network structures and probabilistic directed acyclic graph (DAG) models, require access to individual participant data to directly model causal probabilities, assess the strength and direction of potential biases, and use counterfactuals or negative controls; these methods may therefore not be accessible to secondary reviewers or risk assessors. Although more limited, DAGs can also be used to support summary-level quantitative bias adjustments, such as *E*-values, that capture the range of uncertainties and plausible outcomes. Application of these methods will further inform the impact of residual biases and level of confidence in the evidence available for evaluating causality. DAGs are commonly applied in epidemiological analyses to show interrelated relationships between the hypothesized exposure-response pathway and confounders, effect modifiers, and other variables of interest. The USEPA recently presented on the use of DAGs for causal analysis in environmental assessments (Carriger, 2021) and has published on their use for cumulative risk assessment (Brewer *et al.*, 2017); however, no such methods were employed in the Agency’s most recent evaluation of formaldehyde.

Critically, typical interpretations of quantitative exposure-response models falsely assume a causal relationship where exposure is directly relatable to a change in average response. However, these assumptions may not adequately account for direct or indirect causal effects of other variables (such as smoking), which could lead to inaccurate quantification of individual and population-level risks (Cox, 2023). Analyses have shown significantly increased risks of LHP cancers attributable to smoking, and these effects may be of sufficient magnitude to indicate that the observed associations between formaldehyde and LHP cancers are likely to be attributable to bias (Brown *et al.*, 1992; Fircanis *et al.*, 2014; Musselman *et al.*, 2013; Shi *et al.*, 2019; Ugai *et al.*, 2017). Although some degree of residual confounding is expected, a lack of adjustment or consideration for confounding or modifying factors that are strongly associated with the outcome (such as smoking) precludes assessors from confidently interpreting observed associations between exposure and outcome as true representations of exposure risk. Given the likelihood that such biases could potentially explain the inconsistent findings within the human evidence stream, these methods could provide both qualitative and quantitative information to characterize the impact of these systemic biases and thus better characterize the uncertainties in these observational data. Utilization of these methods would also be helpful in reducing the use of animals to better incorporate observational data in causation analyses and risk assessment.

## Conclusions

The inconsistent associations reported in some worker studies (a subset of all available evidence) were not considered to be demonstrative of a causal relationship between formaldehyde exposures and LHPs when integrated with the totality of the epidemiological evidence (including meta-analyses), toxicological data, and considerations of biological plausibility. The RoB assessment herein, along with the integration of other evidence streams, demonstrated that the inconsistent findings are likely to be spurious and explained by residual bias rather than being indicative of a causal relationship.

## Supplementary Material

kfae039_Supplementary_Data
