# Supplementary material for: Assessment of associations between inhaled formaldehyde and lymphohematopoietic cancer through the integration of epidemiological and toxicological evidence with biological plausibility
Source: Toxicol Sci. 2024 Mar 28;199(2):172–93. doi: 10.1093/toxsci/kfae039 (PMC11131035; doi:10.1093/toxsci/kfae039)
Supplement: kfae039_Supplementary_Data [file kfae039_supplementary_data.zip › kfae039_Supplementary_Data/toxsci-23-0478-File007.docx]

**Table S1**. Eligibility criteria for inclusion or exclusion of studies in this assessment, as described in OSF protocol (Fitch et al., 2023)

| **Element** | **Inclusion Criteria** | **Exclusion Criteria** |
| --- | --- | --- |
| *Population (P)* | - Humans (all populations) - *In vivo* Experimental animal models relevant to humans (Mammalian only, e.g., rats, mice, monkeys) | - Non-mammalian models (e.g., zebrafish, chickens) - *In vitro* assays |
| *Exposures (E)* | - In epidemiology studies: formaldehyde or formalin exposure via inhalation estimated qualitatively or measured quantitatively via personal or area samples - In experimental animal studies: controlled inhalational exposures measured via external metric - Co-exposures in occupational studies as long as formaldehyde and formalin are the primary exposures^1^. | - Formaldehyde via other routes of exposure - Co-exposures to other chemicals in which formaldehyde or formalin are not the primary exposure in epidemiological studies - Any co-exposure in an experimental animal study - Biomonitoring or other internal exposure metrics^2^ |
| *Comparator (C)* | - Vehicle control, low/no exposure estimate | - Study designs that do not include a vehicle or unexposed control group |
| *Outcome(s)(O)* | Measures of LHP malignancies, including^3^: Hodgkin lymphoma (ICD-9, 201; ICD-10, C81), multiple myeloma (ICD-9, 203; ICD-10, C90), lymphoid leukemia (ICD-9, 204; ICD-10, C91), myeloid leukemia (ICD-9, 205; ICD-10, C92), monocytic leukemia (ICD-9, 206; ICD-10, C93), reported either as incidence or mortality based on medical records | - Sinonasal, nasopharynx, lung, or other cancers - Non-specific LHPs, including non-Hodgkin lymphomas and “other” or “unspecified” leukemias^4^ (ICD-9 codes 200, 202, 207, 208; ICD-10 codes C82, C84, C85, C88, C94, C95, C96) - Mechanistic outcomes (i.e., individual studies that inform MoA)^5^ |
| *Study Designs* | - Primary studies/empirical evidence of LHP - Human - Cohort, case-control, or cross-sectional studies - Animal - *in vivo* chronic bioassays^[[1]](#footnote-2)^ - Secondary reviews or assessments of mode of action | - In vitro, ex vivo, and in silico models - Case studies/series - Reviews (narrative or systematic) and meta-analyses^6^ |

^1^ Occupational co-exposures (e.g., dust, phenols, solvents) not independently associated with LHPs are not expected to confound results (USEPA 2022), however these co-exposures should be considered. If formaldehyde is an occupational exposure secondary to other known or potential carcinogens, or chemicals not proven to be unlikely to generate LHP cancers, then the study findings would limited in their utility for assessing the link between formaldehyde, specifically, and LHP malignancies.

^2^ Biomarkers of exposure are expected to be of limited utility for exposure estimation as, to date, no reliable biomarkers for formaldehyde have been identified. ACGIH has not established a No BEI© for formaldehyde. The ATSDR (1999) states that “Attempts have been made to determine if either blood or urinary levels of formaldehyde or formate could be used as potential biomarkers of exposure, but with disappointing results" (p. 230). DNA-protein cross links in white blood cells (Shaham et al. 1996), other DNA adducts, and IgG antibodies in serum (Carraro et al. 1997) are potential biomarkers of both exposure and effect, however their reliability and validity are limited due to intra-personal variability and the inability to differentiate between endogenous and exogenous exposures. Therefore, measurements of exposure based on biomarkers are not considered as reliable for this assessment.

^3^ LHP cancers considered for this evaluation are leukemias, lymphomas and myeloma.

^4^ Per NAS (2011) comments, groupings of diverse LHP cancers with different etiologies and cell origins is not informative for exposure-response or causal evaluations. This is consistent with the USEPA’s approach, in which NHL (“a non-specific grouping of dozens of different lymphomas” for which classification and subtype inclusions have changed over time) is not included in the formal review (EPA 2022a; p. A-667). Due to the lack of specificity in etiology or cell type and the lack of significant associations between formaldehyde inhalation and NHLs in prior reviews (e.g., Catalani et al. 2019), NHLs are excluded from this analysis.

^5^ Individual studies reporting mechanistic data, or data informing MoA, will be excluded from the RoB assessment and quantitative assessments but retained for contextual considerations. Evaluations of MoA will be retained and relied upon for assessment of biological plausibility.

^6^ Reviews and meta-analyses will be excluded but reviewed for contextual considerations and as part of the literature identification phase. Studies identified by these publications will be cross-checked with studies included in the title/abstract review of this effort.

------------

**Table S2 -**  Summary of topic-specific considerations determined *a priori* for the application of the OHAT RoB to address formaldehyde-specific considerations (see Supplemental Material S5 for comprehensive refinements and guidance)

| **Bias** | **Question** | **Evidence stream*** | **Refinement** |
| --- | --- | --- | --- |
| Selection | 1. Was administered dose or exposure level adequately randomized? | Experimental animal | None |
| Selection | 1. Was allocation to study groups adequately concealed? | Experimental animal | None |
| Selection | 1. Did selection of study participants result in appropriate comparison groups? | Human observational | Clarifications of bias characterizations. Direct evidence is additionally defined as demographic information showing no differences between exposed and non-exposed groups; if differences are adjusted in analyses, the risk of bias may be lower. |
| Confounding | 1. Did the study design or analysis account for important confounding and modifying variables? (Key Question) | Human observational | Refined to define and account for known confounders and co-variates of interest, including: age, sex, smoking, formalin use (methanol co-exposure), benzene, and ionizing radiation. Ionizing radiation and formalin exposures occur in populations of embalmers (Hauptmann et al., 2009). |
| Performance | 1. Were experimental conditions identical across study groups? (Key Question) | Experimental animal | None |
| Performance | 1. Were the research personnel and human subjects blinded to the study group during the study? | Experimental animal | None |
| Attrition/  Exclusion | 1. Were outcome data complete without attrition or exclusion from analysis? | Experimental animal; human observational | Refined to specify acceptable rates of loss-to-follow-up or attrition in epidemiological studies.  No refinements to experimental animal studies. |
| Detection | 1. Can we be confident in the exposure characterization? (Key Question) | Experimental animal; human observational | Refined to consider both: 1) quality of quantitative measurements of exposure through incorporation of methods reporting and validation requirements and 2) applicability of measurements for exposure-response evaluation, including reports of exposure duration and frequency of exposure.  No refinements to experimental animal studies. |
| Detection | 1. Can we be confident in the outcome assessment? (Key Question) | Experimental animal; human observational | Refined to consideration both: 1) implications of use of mortality versus incidence of outcome, and 2) confidence in the method of ascertainment (e.g., confirmation with medical records or physician diagnosis)  No refinements to experimental animal studies. |
| Selective Reporting | 1. Were all measured outcomes reported? | Experimental animal; human observational | None |

*Evidence stream in this context refers to experimental animal studies or observational human studies.

1. Mammalian chronic bioassays are traditionally defined as having a lifetime (i.e., 2-year duration) exposure. Per OECD 452 guidelines, a chronic toxicity study should have a minimum of 12 months exposure. [↑](#footnote-ref-2)
